# An automated graphics tool for comparative genomics: the Coulson plot generator

**DOI:** 10.1186/1471-2105-14-141

**Published:** 2013-04-27

**Authors:** Helen I Field, Richard MR Coulson, Mark C Field

**Affiliations:** 1 LGC Genomics Ltd, Pindar Road, Hoddesdon, Hertfordshire EN11 0WZ, UK; 2 Cambridge Institute for Medical Research, University of Cambridge, Cambridge CB2 0XY, UK; 3 Department of Pathology, University of Cambridge, Tennis Court Road, Cambridge CB2 1QP, UK

## Abstract

**Background:**

Comparative analysis is an essential component to biology. When applied to genomics for example, analysis may require comparisons between the predicted presence and absence of genes in a group of genomes under consideration. Frequently, genes can be grouped into small categories based on functional criteria, for example membership of a multimeric complex, participation in a metabolic or signaling pathway or shared sequence features and/or paralogy. These patterns of retention and loss are highly informative for the prediction of function, and hence possible biological context, and can provide great insights into the evolutionary history of cellular functions. However, representation of such information in a standard spreadsheet is a poor visual means from which to extract patterns within a dataset.

**Results:**

We devised the Coulson Plot, a new graphical representation that exploits a matrix of pie charts to display comparative genomics data. Each pie is used to describe a complex or process from a separate taxon, and is divided into sectors corresponding to the number of proteins (subunits) in a complex/process. The predicted presence or absence of proteins in each complex are delineated by occupancy of a given sector; this format is visually highly accessible and makes pattern recognition rapid and reliable. A key to the identity of each subunit, plus hierarchical naming of taxa and coloring are included. A java-based application, the Coulson plot generator (CPG) automates graphic production, with a tab or comma-delineated text file as input and generating an editable portable document format or svg file.

**Conclusions:**

CPG software may be used to rapidly convert spreadsheet data to a graphical matrix pie chart format. The representation essentially retains all of the information from the spreadsheet but presents a graphically rich format making comparisons and identification of patterns significantly clearer. While the Coulson plot format is highly useful in comparative genomics, its original purpose, the software can be used to visualize any dataset where entity occupancy is compared between different classes.

**Availability:**

CPG software is available at sourceforge http://sourceforge.net/projects/coulson and http://dl.dropbox.com/u/6701906/Web/Sites/Labsite/CPG.html

## Background

With a rapidly growing database of completed genomes and consequential improvements to the reconstruction of deep and broad phylogenetic relationships, it has become possible to consider the molecular origins of many complex cellular systems. Such analyses can reveal deep relationships between cellular functions, identify lineage-specific features and uncover evolutionary mechanisms [1-5], and are important in the identification of, for example, pathogen-associated gene products, with potential for therapeutic intervention, as well as in attempts to understand how such systems arose. Further, falling costs of nucleotide sequencing are providing opportunities to generate genome sequences from even hard to culture organisms, making analysis of function in these taxa possible through comparison with tractable organisms. In short, the need to present comparative data is highly pressing and likely to remain an issue for some time.

While it is now comparatively trivial to generate vast datasets containing 100s to 1000s of query results using BLAST, HMMer and other sequence-based algorithms [6-10] these data constitute essentially gene lists, which only have value when processed and presented coherently [5,11-16]. The major biological added value within such analyses is the ability to rapidly compare the distributions of genes between multiple biological processes, i.e. protein complexes and pathways, and also across many taxa. This is quite challenging as these datasets can contain may hundreds/thousands of gene calls, and unless these data are represented graphically and in an easily comprehended manner, patterns are difficult to observe. In particular, spreadsheets do not lend themselves to browsing and fragmenting datasets into subgroups to reduce data complexity often removes much valuable comparative information. Production of comparison figures from developing datasets (works in progress) are invaluable during dataset production, and even for making decisions and developing hypotheses, but manual production of figures on the fly is unfeasible.

To address these needs we devised the Coulson plot, a matrix of colorized pie charts and which displays information in a clustered format, together with hierarchical taxonomic labels and a key to individual gene products. This plot we, and others, have used in multiple publications and which we have found to be highly useful and accessible to readers of these reports [3,17-24]. However, the manual construction of these plots is time consuming and, with hundreds of elements, error prone, and which precludes *on-the-fly* plots and possibly wider adoption of the format. Hence, to facilitate generic/automated production and adoption of the plot we developed a platform-independent application, the Coulson plot generator (CPG), to draw Coulson plots from structured data that uses standard spreadsheet file formats as input. CPG should be accessible to the vast majority of workers with only rudimentary computing skills and requires minimal post-plot manipulations to generate publication quality plots of considerable complexity.

## Implementation

### Graphical concept

**Figure 1:**
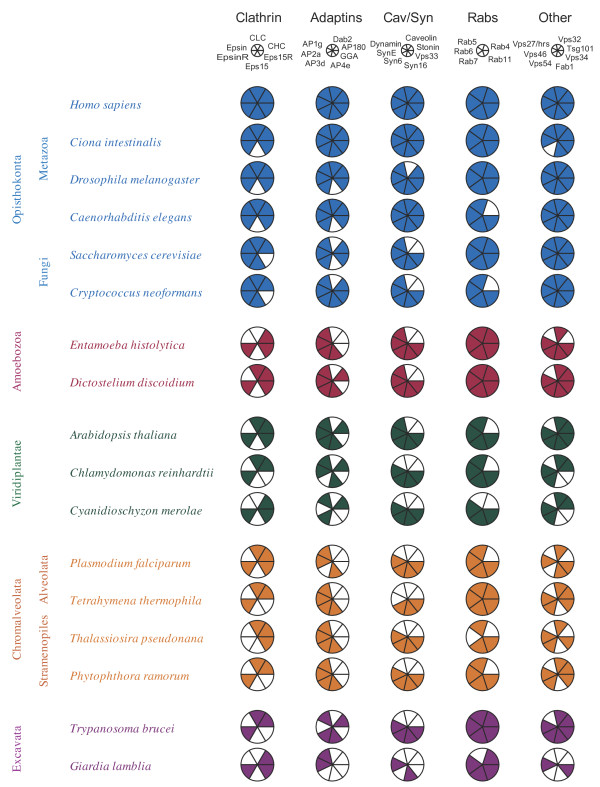
**A Coulson plot as generated by the CPG java application.** A collection of data corresponding to complexes or functional systems within the eukaryotic trafficking pathway have been used to illustrate principle aspects of the plot format. At left supergroup designations and taxa (species) are colored by supergroup *sensu* Adl (Adl et al. 2004). At the top is a key that bears the complex name and a smaller gray pie where the sectors are labelled. Below this, in rows, are the individual pies representing gene distributions for each species, with colors that match the taxon supergroup colors. This format allows easy detection of patterns in the data, for example the absence of Vps27 orthologs from all taxa other than Opisthokonta (rightmost pie, 10 to 11o’clock). All plot and annotation elements have been created exclusively by the application. Importantly, CPG allows plots to be produced during gathering of datasets in a rapid manner before production of camera ready graphics. Minor adjustments, including additional spacing between supergroup rows has been introduced after CPG production for clarity, and taxon names italicized; this has been done in a third party vector graphics package that can edit pdf files.

We considered many of the formats commonly used in the published literature for the display of comparative genomic data, and found these frequently too complex or inelegant for the presentation of data in a manner that retains as much biological information as possible. Specifically, simple spreadsheets or dot plots are either difficult to read or lose information concerning complexes, which is especially critical to understanding evolutionary processes. In many instances dot blots also become very large, with moire effects and other issues emerging. Hence we designed a more sophisticated format that retains functional groupings, provides colors as keys to taxonomic relationships and also provides a key to subunit identity (Figure 1). We consider that the Coulson format retains more information than dot plots and provides this in an attractive and easy to comprehend manner.

### Algorithm design

**Figure 2:**
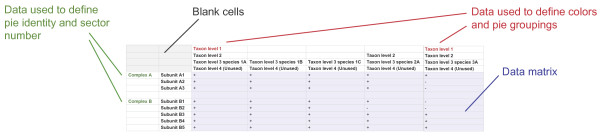
**Data as imported into a CPG application table.** Data are most conveniently assembled in a spreadsheet and then saved as tabbed text or .csv format. The left-most two columns are the complex categories, with the overall complex name in column one (green) and the subunits in column two; the number of subunits in column two is used to provide the correct number of sectors in the pie chart. A space/empty cell separates each system. The top four rows contain the taxonomic information. The top level is used to define colors for groups of pies (red), and the remaining are for labeling only. In the example in Figure 1 the fourth row is unused. Below these rows is the data matrix (blue). All cells corresponding to a subunit must be filled and empty cells are not allowed.

**Figure 3:**
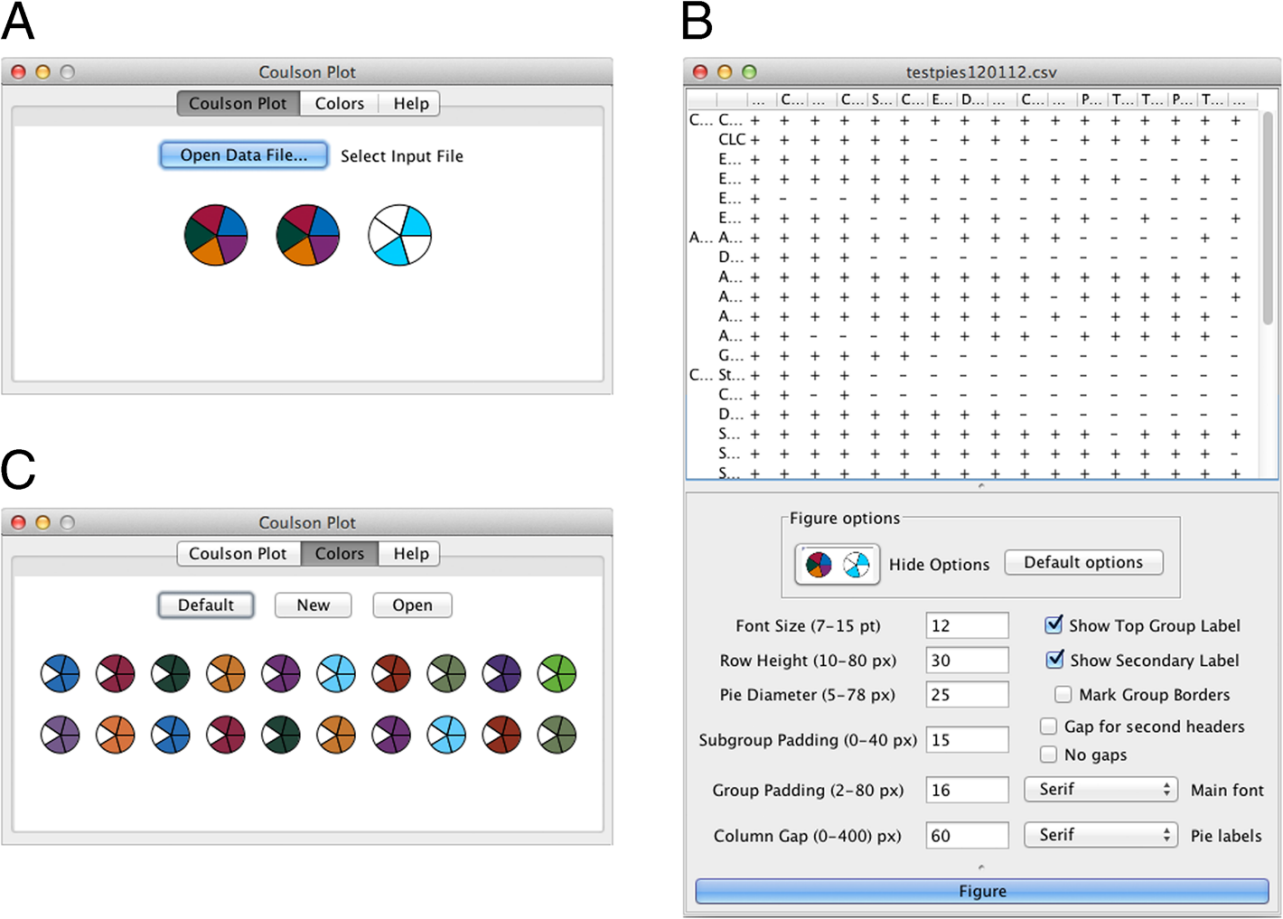
**CPG application windows and dialogs.** Panel **A**: On launch CPG offers three basic options, color selection, data file selection and a log/help option. A fourth tab which logs interaction with the system is optionally available (Preferences). Panel **B**: Selection of either a default color set or production of a custom set is done using a color picker is facilitated in the ‘Colors’ window. Color selections can be saved as text files to any directory for later reuse. Options to change fontsize, pie size and pie spacing, as well as spacing between systems, are provided. Panel **C**: The plot window. The parsed data are shown at top, allowing the user to visually validate the data prior to running the plot routine. Options to change font size, for precise control of spacing and sizes of individual pie charts are provided. Clicking on ‘Plot’ generates the graphical output.

Originally, we generated Coulson plots using Perl with individual data structures for each diagram, with individual programming for each diagram, requiring considerable time and programming expertise to produce a basic figure [3]. In addition this is also potentially an error prone process. A Coulson plot generator (CPG) application was written as an open source, stand alone program developed in Java using Eclipse (http://www.eclipse.org) to execute on any machine running a Java Virtual Machine (v1.5.0 or greater). CPG takes as input a comma separated (.csv) text file of binary data recording subunit occupancy in multiple systems (Figure 2). Systems, e.g. multi-protein complexes, are labelled in the first column, with subunits in the second. Then the data itself (+/−) begins in subsequent columns. Use of either ‘+’ and ‘-’ or ‘1’ and ‘0’ for data occupancy are supported. Only one protein name is required per list of subunits, and column one is occupied only at the position of the first subunit of each group. The input table uses the four top rows and two columns for labeling. Kingdom or supergroup names [25] fall in rows one and two, while species names fall on row three, which must all be occupied and no gaps are permitted. The fourth row can be left unused, omitted completely or utilised for additional taxonomic annotation if desired. The input table uses the first two columns for labeling, with a protein/entity name in column one, and subunit names in column two. Only one protein name is required per list of subunits, and column one is occupied only at the position of the first subunit.

CPG parses the input file and breaks it up into an array of tab-delimited strings. These are processed and displayed as a table, where diagram settings can then be selected (Figure 3B). For creating the figure, the CPG algorithm takes each row, representing the occupancy data across species for one single subunit of a protein and tracks which protein/entity the subunit belongs to. When all the subunits have been collected for a protein/entity, a row of pies is plotted. Species can be separated by Kingdom or supergroup in the same way, and colors are allocated for each supergroup. Pie data are stored in vectors containing 1 or 0 to show pie occupancy which is converted into a graphic (Figures 1 and 3). The collection of graphics for all the pies are laid out with labels. The number of segments for each pie depends on the number of rows associated with a complex: the number of pies depends on the number of species (indicated by columns). Once plotted, the image is stored in memory and resized by zooming in or out, and may be saved in various image formats as well as editable images (svg in PDF or svg format).

### Program operation

The CPG application opens with three tabs (Figure 3A). The first allows the user to select an input data file, the second, to choose custom pie colors, and the third tab provides the Help/Manual and change log (and licensing). The fourth tab provides a process log, and information to assist with input file formatting (appropriate error messages if your input is not acceptable). The 'Plot' button is not enabled unless the input is correct; clicking on 'Plot' generates the figure. By returning to the first, tabbed window, multiple plots may be created from different inputs, and different versions of a figure may be created from the second window and viewed all together. A default color set is supplied (text file and hard coded) (Figure 3B). After selecting an input file, CPG will parse the data and if successful it will convert the data to a table (Figure 3C). Clicking ‘Figure’ will display a Coulson plot of the data in a new scrollable window. An example dataset used for testing is shown (Figure 3B) from which a small portion was taken for early development. The text file was produced using Microsoft Excel, with data entry in the table as described (Figure 2). Data from Excel were exported as comma separated files (.csv). The output file is an editable PDF or SVG file which can be opened and manipulated with Inkscape (http://inkscape.org/) or Adobe Illustrator (http://www.adobe.com/products/illustrator.html?promoid=KAUCB). We selected this option as more efficient than attempting to build sophisticated editing tools into CPG as the precise choices and requirements of users and datasets are difficult to predict. This follows a similar philosophy to FigTree, a popular phylogenetics tree graphics package which also generates editable graphics requiring a small amount of finessing prior to publication (http://tree.bio.ed.ac.uk/software/figtree/). A Coulson plot with more than 200 pies can be produced satisfactorily.

## Results

We developed the Coulson plot to display and compare data on gene representation grouped by gene product complex or pathway membership and to display this information across multiple taxa (Figure 1). An array of gene product components from multiple species with each complex is displayed as a pie chart comprising a variable number of components (sectors), the number of which matches the number of protein subunits in a functional complex, process (i.e. pathway) or other functional group. Pie charts are arranged by phylogenetic hierarchy to allow evaluation of evolutionary trends and the rapid identification of gene losses, specializations or expansions. Several such systems may be compared, so that an array of systems is represented for each species. Using colors, it is possible to separate groups of systems with excellent visual clarity.

**Figure 4:**
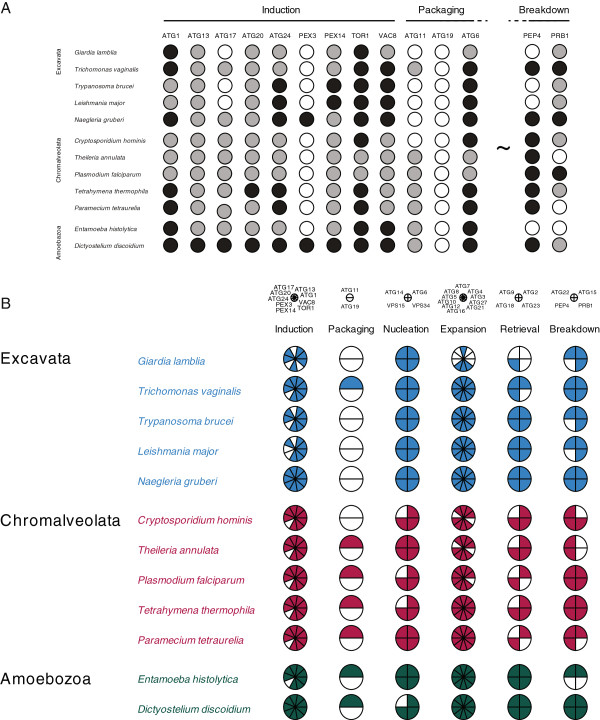
**Two distinct display formats that can be produced using CPG, based on the autophagy pathway in select protists.** Panel **A**: A traditional three-state dot plot representation, similar to that presented in the original publication [25]. White designates not found or absent, black a confident assignment, and gray a lower confidence assignment, in this case lacking phylogenetic support. The plot has some appeal, is clean, but is also large, and lacks functional groupings, making assessment of differential levels of occupancy of the distinct complexes or processes within the autophagy pathway difficult to comprehend, while the plot is also not very compact. Tilda and dotted lines indicate many columns omitted for space reasons. Panel **B**: Standard CPG format, with each complex represented as a single pie, and the higher order taxon membership colorized. Note that the plot is considerably more compact, and complex occupancy and/or subunit retention clear, even for complexes containing many subunits. Data are taken from ref 24 for illustrative purposes only. Both figures generated with CPG with manipulations in Adobe Illustrator. Note that three state occupancy is not allowed in CPG at present so that gray circles in panel A were manually colored.

One of the more flexible aspects of the CGP is that the user can decide quickly how best to group data. For example, complexes or pathways with many components may be difficult to visualize in the individual pie charts, with the result that clarity is lost. However, CPG allows pies to be set up that have only one subunit, for example mimicking the more standard dot blot format, or to subdivide the data into subcomplexes with biological relevance, to improve clarity or increase the amount of data that may be logically compared (Figure 4). A second flexible feature is the ability to manually edit the plot to improve spacing, add additional annotation and change fonts, which allows the user to control the graphic and maintain consistency with additional elements in a figure. Overall, we have found that CPG improves workflow and reduces data transcription errors. Finally, the program is lightweight, making minimal impact on CPU resources and runs without issue on most major platforms.

## Conclusions

We have found the Coulson plot to be highly valuable for presentations of comparative genomic data, and that the lucid display of patterns within datasets more than offset the time required to manually produce these plots. However, we are aware that the skills required and potentially the effort needed acted as a barrier to adoption of a broadly potentially useful graphing format, and which is not available as part of commercial graphing packages as far as we are aware. We therefore developed a plotting tool that manages the vast majority of the plot functionality, leaving the user a format that can be subjected to final editing as appropriate for individual requirements.

A great many datasets have been used to test CPG [3,17-24]. We find the software is stable on OS X (10.5.8 to 10.8.2), Microsoft Windows (XP, 7 and 8) and multiple versions of Linux. Creation of .csv output files from Microsoft Excel, Apple Numbers or open source office suites that can be read by CPG is routine, and the PDF and SVG output successfully imported to Adobe Illustrator or Inkscape as an editable graphic. A diagram with more than 200 pies and over 600 individual elements can be routinely produced, allowing publication quality figures to be generated in one hour. The ability to rapidly generate plots from dissimilar datasets *on-the-fly*, allowing hypothesis-driven composition of datasets, is a distinct advantage, and we hope that the Coulson plot will become a more generally exploited format, and that the use of this plot beyond comparative genomics will be facilitated with the provision of CPG.

## Availability and requirements

CPG is a Java application and requires Java 1.5.0 or higher for the JVM. CPG source code and binaries are available from sourceforge: http://sourceforge.net/projects/coulson as a jar file or disc image for Mac OS X. Project home page: http://dl.dropbox.com/u/6701906/Web/Sites/Labsite/CPG.html and http://sourceforge.net/projects/coulson. The software is licensed under GNU Artistic license 2.0.

## Competing interests

The authors declare that they have no competing interests.

## Authors’ contributions

HIF created and tested the software. MCF identified the need and had input on software design and features. RMRC designed the original Coulson plot graphic format. All authors participated in the writing and approval of the manuscript and β-testing of the software. All authors read and approved the final manuscript.
